# Economic burden of hospitalization for Chinese children with chronic kidney disease: a comparison between patients with and without infection

**DOI:** 10.3389/fped.2025.1554929

**Published:** 2025-04-04

**Authors:** Xin Shi, Lixia Li, Yuxing Zhu, Xun Liu, Yikun Mou, Lei Guo

**Affiliations:** Department of Pediatrics, Third Affiliated Hospital of Sun Yat-sen University, Guangzhou, China

**Keywords:** chronic kidney disease, children, hospitalization costs, infection, urine protein

## Abstract

**Objective:**

To assess hospitalization costs in pediatric chronic kidney disease (CKD) patients, compare the economic burden between those with and without infections, and identify key factors influencing these costs, emphasizing the significant financial impact on families and healthcare systems.

**Methods:**

This retrospective analysis included pediatric patients with CKD hospitalized between May 2011 and April 2020. Clinical characteristics, including demographics, etiology, urinary protein level, estimated glomerular filtration rate, and CKD stage, were analyzed. Hospitalization costs were compared between groups with and without infection using appropriate statistical methods.

**Results:**

Among 721 pediatric CKD patients included in this study, 388 had primary kidney disease and 333 had secondary kidney disease. Patients in the infection group had significantly higher urine protein levels, longer hospital stays, and higher total hospital fees than those without infection (all *P* < 0.05). In the primary kidney disease cohort, patients aged 14–18 years incurred the highest costs (16,706 CNY, *P* = 0.009), while those with 1 + urine protein levels had expenses averaging 29,813 CNY (*P* = 0.035). In the secondary kidney disease cohort, the 3 + urine protein group had the highest costs (62,841 CNY, *P* < 0.001). Multiple linear regression identified age, urine protein level, and length of hospital stay as significant cost determinants. Patients with infection in the secondary kidney disease cohort had an average additional expenditure of 13,572.55 CNY compared to those without infection (*P* = 0.001).

**Conclusion:**

This study highlights the economic burden of infection during pediatric CKD hospitalization, emphasizing the need for effective infection management strategies to reduce financial strain and improve outcomes.

## 1 Introduction

Chronic kidney disease (CKD) in children has emerged as a significant global health issue, affecting an estimated 15–74.7 cases per million children globally and posing considerable risks to pediatric populations. CKD is defined as an abnormality in kidney structure or function that lasts more than 3 months (1, 2). Additionally, this condition can persist into adulthood, impacting up to 10%–15% of the adult population globally (2, 3). Recent epidemiological studies have reported a rising prevalence of CKD among children, positioning it as one of the most serious threats to pediatric health today (4). The etiology of pediatric CKD is multifaceted, encompassing primary kidney diseases ·like congenital anomalies and secondary conditions arising from systemic illnesses, such as systemic lupus erythematosus and Henoch-Schönlein purpura. This complexity leads to varied rates of disease progression and distinct hospitalization patterns, placing considerable strain on healthcare resources and underscoring the urgent need for effective management strategies (5, 6).

The increasing incidence of pediatric CKD has profound implications for healthcare systems, particularly concerning the economic burdens borne by families and healthcare providers. The costs associated with managing CKD in children can be substantial, often resulting in significant financial distress for families navigating the complexities of long-term treatment and care (7, 8). Unlike adult CKD patients, who commonly experience complications such as hypertension and diabetes, children with CKD are at risk for a greater variety of complications, including hypertension, anemia, electrolyte imbalance, and increased susceptibility to infection, which have become more prevalent, especially during the coronavirus disease 2019 (COVID-19) pandemic (9). Infections not only complicate the clinical management of CKD but also substantially increase hospitalization costs, exacerbating the economic burden on both families and the healthcare system. Despite the recognized significance of these complications, comprehensive studies addressing their impact on hospitalization costs for pediatric CKD patients are lacking.

As the understanding of pediatric CKD evolves, the results of economic analyses must be integrated into clinical care and healthcare policies to effectively address the needs of these patients. While the existing literature has primarily focused on the relationship between specific comorbidities and CKD outcomes, the broader context of hospitalization expenses has been largely overlooked (10, 11). The present study aimed to analyze hospitalization costs and their components in a cohort of 721 pediatric CKD patients with a systematic investigation of the interplay between common infections and hospitalization outcomes. By providing insights into the economic burden associated with pediatric CKD, the findings may inform targeted interventions aimed at reducing hospitalization costs and improving patient outcomes, ultimately guiding healthcare providers to make informed decisions regarding resource allocation and management strategies for this vulnerable population.

## 2 Materials and methods

### 2.1 Study design

This study was conducted using the renal disease database of the Third Affiliated Hospital of Sun Yat-sen University. A retrospective analysis was conducted on hospitalization costs for pediatric patients under 18 years of age diagnosed with CKD. Additionally, the study compared the hospitalization costs between CKD patients who did or did not experience infection.

The study received approval from the Ethics Committee of the Third Affiliated Hospital of Sun Yat-sen University [approval number: (2019)02-427-01] and complied with the principles outlined in the Declaration of Helsinki. Informed written consent was obtained from the patients' parents or guardians.

### 2.2 Study population

This study included pediatric patients aged 18 years or younger who were hospitalized at the Third Affiliated Hospital of Sun Yat-sen University between May 2011 and April 2020. A total of 721 patients were included based on the definitions and criteria for CKD.

Inclusion criteria: According to the 2012 clinical practice guidelines from Kidney Disease: Improving Global Outcomes (KDIGO), CKD was defined as abnormalities in kidney structure or function lasting for more than 3 months, with health implications, and meeting at least one of the following criteria (12): (1) proteinuria (urinary protein excretion rate ≥30 mg/24 h; urine protein-to-creatinine ratio ≥30 mg/g); (2) urinary sediment abnormalities; (3) electrolyte and other abnormalities due to tubular disorders; (4) histological abnormalities; (5) structural abnormalities detected by imaging; (6) history of kidney transplantation; (7) decreased glomerular filtration rate (GFR) for at least 3 months; and (8) estimated glomerular filtration rate (eGFR) < 60 ml/min·1.73 m².

Exclusion criteria: (1) acute kidney injury at time of hospitalization; (2) significant comorbidities affecting kidney function; (3) recent exposure to nephrotoxic agents; and (4) incomplete medical records or insufficient lab data.

### 2.3 Clinical data collection

To assess the factors influencing hospitalization costs for pediatric patients with CKD, data for a variety of variables were collected from the renal disease database. Key demographic variables, such as gender and age, were recorded for analysis of their impact on treatment and costs. Additionally, the etiology of CKD was documented for use in investigating the underlying causes of the disease.

Clinical metrics included disease history, urinary protein level, eGFR, CKD stage, and complications such as the site and severity of infections, all aimed at evaluating disease severity and progression. The length of hospitalization also was recorded for analysis of its impact on overall costs. On qualitative urine protein testing (indicator protein error method), the results were categorized as negative, trace, 1+, 2+, and 3+, corresponding to no proteinuria, trace, mild, moderate, and heavy proteinuria, respectively. CKD was staged based on the eGFR as follows: eGFR ≥90 ml/min·1.73 m² as stage 1, corresponding to normal kidney function with kidney damage; eGFR 60–89 ml/min·1.73 m² as stage 2, reflecting a mild decrease in kidney function; eGFR 30–59 ml/min·1.73 m² as stage 3, indicating moderate impairment; eGFR 15–29 ml/min·1.73 m² as stage 4, indicating severe kidney dysfunction; and eGFR <15 ml/min·1.73 m² as stage 5, representing end-stage renal disease.

The economic burden data included all in-hospital fees, with detailed records of medical insurance expenses and out-of-pocket costs incurred by families. Specific fees related to diagnosis, treatment, nursing care, laboratory tests, imaging, and clinical assessments were documented. Additionally, costs associated with surgical treatments, expenses for Western and traditional Chinese medicine, and material costs were recorded. This comprehensive data collection was assembled for use in identifying key cost drivers, with the goal of informing strategies to improve care while alleviating the financial burden on families managing pediatric CKD.

### 2.4 Statistical analysis

All statistical analyses were conducted using Stata/SE version 17.0 (StataCorp LP, College Station, TX, USA). The normality of all continuous variables was assessed using the Kolmogorov–Smirnov test. As none of the continuous variables met the normality assumption, they were reported using median and interquartile range (IQR, PR25, PR75) values. Comparisons between patients with and without infection were conducted using the Mann–Whitney *U* test. Categorical variables were presented as counts and percentages, and comparisons were made using the chi-square test or Fisher's exact test (for expected values ≤5). The analysis of total hospitalization costs was also stratified by age group and urine protein level, with intergroup comparisons performed using one-way analysis of variance (ANOVA) followed by Fisher's least significant difference (LSD) *post-hoc* test. A multiple linear regression model was utilized to investigate the linear relationship between overall infection and total hospitalization cost, with adjustments for clinical characteristics as covariates. Statistical significance was defined as *P* < 0.05 for all tests, with a two-tailed approach.

## 3 Results

### 3.1 Patient's clinical characteristics and medical costs

The study population included a total of 721 pediatric patients with CKD, categorized into two distinct cohorts: those with primary kidney disease (*n* = 388) and those with secondary kidney disease (*n* = 333). In the primary kidney disease cohort, 276 patients (71.13%) were male and 112 (28.87%) were female, with a median age of 12 years (IQR: 5.25–16 years). The secondary kidney disease cohort comprised 66 males (19.82%) and 267 females (80.18%), with a median age of 15 years (IQR: 13–17 years).

Table 1 presents comparisons of the clinical characteristics and medical costs between CKD patients with and without infection from the primary kidney disease cohort. As indicated, compared to patients without infection, those who experienced infection were relatively younger and had a lower prevalence of congenital anomalies of the kidney and urinary tract (CAKUT). However, the patients with infection had higher urine protein levels, and longer hospital stays. In terms of medical costs, patients in the infection group had significantly higher in-hospital total fees, out-of-pocket fees, medical services fees, therapeutic procedure fees, nursing fees, laboratory diagnosis fees, non-surgical treatment fees, Western drug fees, antibacterial drug fees, and treatment materials fees (all *P* < 0.05). Despite these elevated costs, the infected patients had a relatively lower average daily cost, likely attributable to their extended hospital stays (*P* = 0.025).

**Table 1:** Comparison of clinical characteristics and medical costs between patients with or without infection within the primary kidney disease cohort.

Parameters	Infection		All (*n* = 388)	*P*
	No (*n* = 207)	Yes (*n* = 181)		
Age, years	14 (7, 17)	10 (4, 15)	12 (5.25, 16)	<0.001
Age by group, *n* (%)				0.002
≤5 years	41 (19.81%)	56 (30.94%)	97 (25.00%)	
6–13 years	57 (27.54%)	61 (33.70%)	118 (30.41%)	
14–18 years	109 (52.66%)	64 (35.36%)	173 (44.59%)	
Sex, *n* (%)				0.536
Male	150 (72.46%)	126 (69.61%)	276 (71.13%)	
Female	57 (27.54%)	55 (30.39%)	112 (28.87%)	
Disease history, *n* (%)				
Hyperuricemia	12 (5.80%)	6 (3.31%)	18 (4.64%)	0.246
Anemia	15 (7.25%)	15 (8.29%)	30 (7.73%)	0.702
CAKUT	57 (27.54%)	34 (18.78%)	91 (23.45%)	0.042
Tumor	11 (5.31%)	6 (3.31%)	17 (4.38%)	0.337
Mental disorder	0	1 (0.55%)	1 (0.26%)	0.466
Urine protein level, *n* (%)				0.002
Negative	65 (36.31%)	29 (17.47%)	94 (27.25%)	
A small amount	12 (6.70%)	9 (5.42%)	21 (6.09%)	
1+	6 (3.35%)	8 (4.82%)	14 (4.06%)	
2+	44 (24.58%)	55 (33.13%)	99 (28.70%)	
3+	52 (29.05%)	65 (39.16%)	117 (33.91%)	
eGFR, ml/min·1.73 m²	138.12 (93.85, 168.99)	148.75 (110.28, 183.57)	143.08 (98.42, 178.73)	0.011
GFR stage	1 (1, 1)	1 (1, 1)	1 (1, 1)	0.196
Hospital stay, days	11 (7, 17)	16 (10, 23)	13 (8, 19)	<0.001
Out-of-pocket fees, *n* (%)				0.058
No	74 (46.25%)	55 (35.71%)	129 (41.08%)	
Yes	86 (53.75%)	99 (64.29%)	185 (58.92%)	
Medical insurance, *n* (%)				0.058
No	86 (53.75%)	99 (64.29%)	185 (58.92%)	
Yes	74 (46.25%)	55 (35.71%)	129 (41.08%)	
Infection type, *n* (%)				
Lower respiratory tract		77 (19.85%)		
Urinary		30 (7.73%)		
Central nervous system		2 (0.52%)		
Digestive tract		21 (5.41%)		
Respiratory		52 (13.40%)		
Uncertain		12 (3.09%)		
Severe disease		0		
Other		8 (2.06%)		
Medical costs (CNY)				
In-hospital total fees	8,257.68 (5,291.70, 12,646.75)	10,182.59 (6,256.17, 17,335.24)	8,746.59 (5,881.36, 14,510.55)	0.005
Out-of-pocket fees	5,552.95 (2,257.27, 8,363.60)	7,247.34 (3,125.19, 13,354.96)	5,953.22 (2,823.07, 10,958.30)	0.006
Medical services fees	517.50 (225, 855.25)	812 (349, 1,182.50)	630 (246.25, 1,001.50)	<0.001
Therapeutic procedure fees	127.06 (8.75, 438.87)	402.50 (110.48, 806.75)	252 (25.79, 616.50)	<0.001
Nursing fees	165.14 (66, 292.25)	234 (95, 389)	189 (78.79, 341.71)	0.002
Pathology diagnosis fees	0 (0, 1,114.25)	0 (0, 185)	0 (0, 459)	0.276
Laboratory diagnosis fees	2,475.02 (993.55, 3,392)	3,204.90 (1,589.25, 4,254)	2,861.50 (1,188.82, 3,815.17)	<0.001
Imaging diagnosis fees	581 (101.20, 1,326.50)	575 (265, 959.50)	575 (190, 1,037.20)	0.804
Clinical diagnosis fees	35 (0, 77.93)	35 (0, 147.86)	35 (0, 132)	0.812
Surgical treatment fees	0 (0, 32.50)	0 (0, 25)	0 (0, 28.75)	0.130
Non-surgical treatment fees	0 (0, 0)	0 (0, 0)	0 (0, 0)	0.003
Western drug fees	1,065.46 (409.07, 2,810.63)	2,207.47 (987.73, 5,439.63)	1,648.11 (634.29, 4,163.39)	<0.001
Antibacterial drug fees	0 (0, 74.28)	168 (0, 787.50)	9.03 (0, 403.65)	<0.001
Patent drug fees	5.11 (0, 134.48)	11.96 (0, 145.39)	10.45 (0, 141.61)	0.590
Herbal drug fees	0 (0, 0)	0 (0, 0)	0 (0, 0)	0.928
Blood product fees	0 (0, 0)	0 (0, 0)	0 (0, 0)	0.375
Examination materials fees	26.18 (0, 50.82)	24.64 (0, 50.82)	24.64 (0, 50.82)	0.466
Treatment materials fees	234.18 (26.11, 574.93)	472.92 (178.24, 857.85)	359.42 (45.88, 778.52)	<0.001
Surgical materials fees	0 (0, 0)	0 (0, 0)	0 (0, 0)	0.101
Average daily cost	878.52 (557.69, 1,317.87)	735.46 (471.68, 1,147.42)	812.59 (516.31, 1,230.68)	0.025

Table 2 presents comparisons of the clinical characteristics and medical costs between CKD patients with and without infection from the secondary kidney disease cohort. As indicated, compared to patients without infection, those who experienced infection had a higher rate of anemia, higher urine protein levels, lower eGFR and GFR stages, and longer hospital stays. With respect to medical costs, while the pathology diagnosis fees, patent drug fees, and herbal drug fees did not differ significantly between the groups, all other cost items were significantly higher in the infection group than in the non-infection group (all *P* < 0.05).

**Table 2:** Comparison of clinical characteristics and medical costs between patients with or without infection within the secondary kidney disease cohort.

Parameters	Infection		All (*n* = 333)	*P*
	No (*n* = 211)	Yes (*n* = 122)		
Age, years	15 (13, 17)	15 (12, 17)	15 (13, 17)	0.534
Age by group, *n* (%)				0.781
≤5 years	6 (2.84%)	5 (4.10%)	11 (3.30%)	
6–13 years	61 (28.91%)	37 (30.33%)	98 (29.43%)	
14–18 years	144 (68.25%)	80 (65.57%)	224 (67.27%)	
Sex, *n* (%)				0.534
Male	44 (20.85%)	22 (18.03%)	66 (19.82%)	
Female	167 (79.15%)	100 (81.97%)	267 (80.18%)	
Disease history, *n* (%)				
Hyperuricemia	6 (2.84%)	3 (2.46%)	9 (2.70%)	1.000
Anemia	14 (6.64%)	16 (13.11%)	30 (9.01%)	0.047
CAKUT	3 (1.42%)	2 (1.64%)	5 (1.50%)	1.000
Tumor	2 (0.95%)	1 (0.82%)	3 (0.90%)	1.000
Mental disorder	0	1 (0.82%)	1 (0.30%)	0.366
Urine protein level, *n* (%)				<0.001
Negative	99 (51.56%)	31 (30.69%)	130 (44.37%)	
A small amount	29 (15.10%)	15 (14.85%)	44 (15.02%)	
1+	17 (8.85%)	10 (9.90%)	27 (9.22%)	
2+	39 (20.31%)	30 (29.70%)	69 (23.55%)	
3+	8 (4.17%)	15 (14.85%)	23 (7.85%)	
eGFR, ml/min·1.73 m²	138.20 (128.16, 152.89)	133.23 (85.13, 145.12)	136.27 (120.46, 151.09)	0.007
GFR stage	1 (1, 1)	1 (1, 2)	1 (1, 1)	<0.001
Hospital stay, days	11 (7, 18)	16 (9, 25.25)	13 (8, 22)	<0.001
Out-of-pocket fees, *n* (%)				0.796
No	74 (45.68%)	52 (47.27%)	126 (46.32%)	
Yes	88 (54.32%)	58 (52.73%)	146 (53.68%)	
Medical insurance, *n* (%)				0.796
No	88 (54.32%)	58 (52.73%)	146 (53.68%)	
Yes	74 (45.68%)	52 (47.27%)	126 (46.32%)	
Infection type, *n* (%)				
Lower respiratory tract		70 (21.02%)		
Urinary		8 (2.40%)		
Central nervous system		7 (2.10%)		
Digestive tract		8 (2.40%)		
Respiratory		21 (6.31%)		
Uncertain		5 (1.50%)		
Severe disease		2 (0.60%)		
Other		15 (4.50%)		
Medical costs (CNY)				
In-hospital total fees	7,921.15 (5,050.19, 12,613.39)	15,910.77 (8,393.92, 42,002.10)	9,531.65 (5,888.06, 18,799.65)	<0.001
Out-of-pocket fees	4,991.52 (1,734.71, 8,752.21)	10,046.36 (4,623.10, 27,341.06)	6,085.29 (2,611.66, 12,944.59)	<0.001
Medical services fees	545 (194.50, 1,005)	1,006 (506, 1,822.50)	632 (303.75, 1,246.75)	<0.001
Therapeutic procedure fees	121.90 (4, 344.77)	503.73 (185.65, 1,514.98)	204 (28.85, 596.05)	<0.001
Nursing fees	148.20 (42.50, 311)	357.64 (150.20, 962.77)	198.99 (85.75, 460.25)	<0.001
Pathology diagnosis fees	0 (0, 0)	0 (0, 0)	0 (0, 0)	0.258
Laboratory diagnosis fees	2,711.50 (1,302.65, 3,825.65)	4,586 (2,790.25, 7,300.43)	3,163 (1,748.55, 4,824.72)	<0.001
Imaging diagnosis fees	762 (0, 1,161.60)	1,276 (663.20, 2,142)	885.50 (253.75, 1,389.30)	<0.001
Clinical diagnosis fees	65 (0, 265.84)	256.28 (35, 799.34)	137.48 (26, 410.80)	<0.001
Surgical treatment fees	0 (0, 25)	0 (0, 32.50)	0 (0, 25)	<0.001
Non-surgical treatment fees	0 (0, 0)	0 (0, 237.50)	0 (0, 0)	<0.001
Western drug fees	1,743.33 (599.52, 3,438.08)	6,131.39 (2,355.41, 21,618.59)	2,414.11 (980.65, 7,122.28)	<0.001
Antibacterial drug fees	0 (0, 0)	554.61 (0, 1,843.81)	0 (0, 573.06)	<0.001
Patent drug fees	0 (0, 64.13)	11.96 (0, 89.02)	0 (0, 66.58)	0.226
Herbal drug fees	0 (0, 0)	0 (0, 0)	0 (0, 0)	0.608
Blood product fees	0 (0, 0)	0 (0, 115)	0 (0, 0)	<0.001
Examination materials fees	0 (0, 26.18)	16.80 (0, 52.36)	0 (0, 26.18)	0.006
Treatment materials fees	199.63 (16.52, 494.10)	576.55 (244.28, 1,563.18)	297.88 (51.70, 900.92)	<0.001
Surgical materials fees	0 (0, 0)	0 (0, 0)	0 (0, 0)	0.030
Average daily cost	741.17 (518.01, 1,030.16)	1,175.09 (814.76, 1,852.90)	880.41 (596.36, 1,274.05)	<0.001

### 3.2 In-hospital total fees for different age groups and urine protein levels

**Figure 1:**
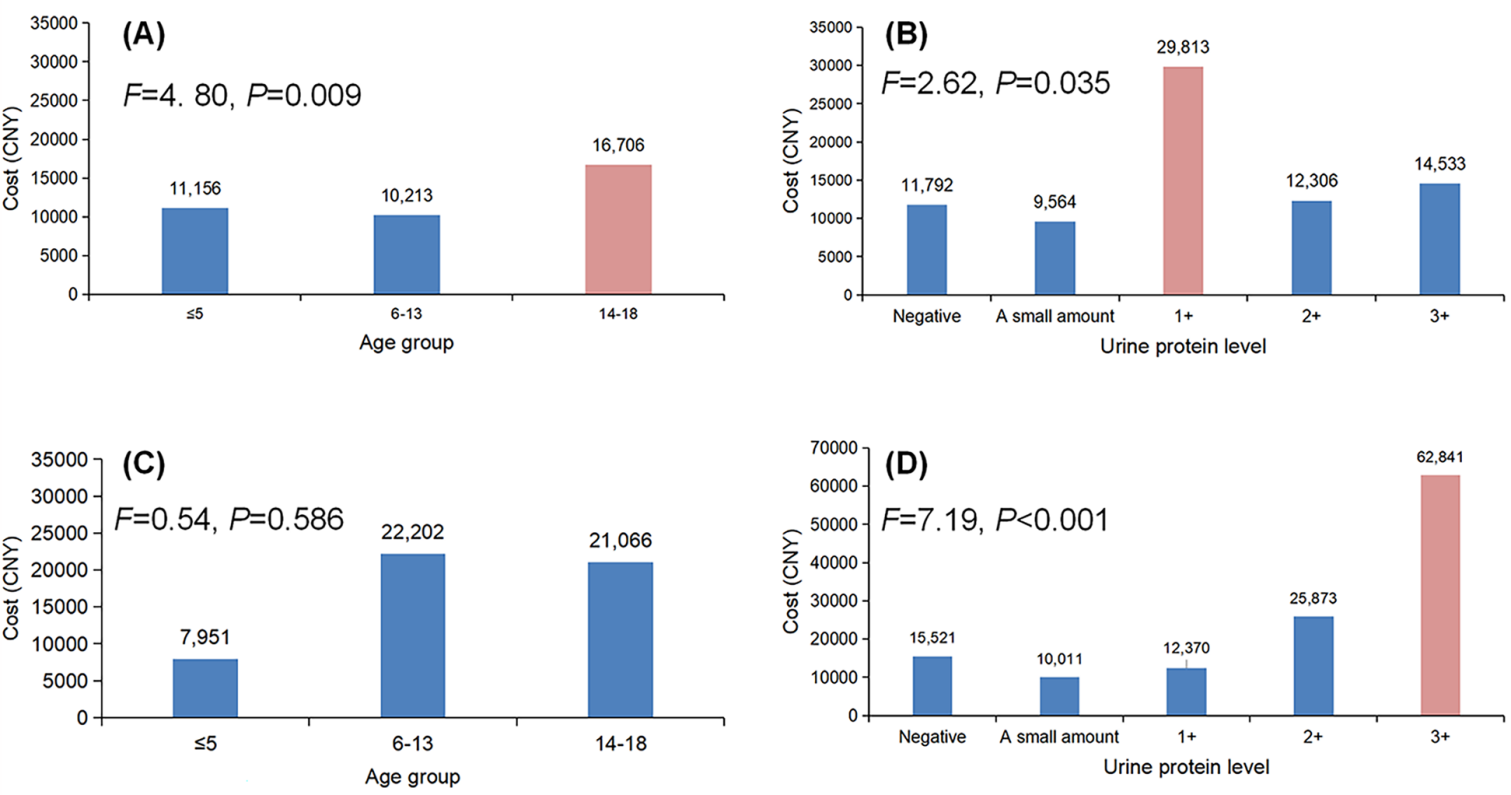
In-hospital total fees of patients stratified by age and urine protein level within the primary and secondary kidney disease cohorts. **(A,B)** Cost comparisons among patients with primary kidney disease stratified by age and urine protein level. **(C,D)** Cost comparisons among patients with secondary kidney disease stratified by age and urine protein level.

Figure 1 illustrates the in-hospital total fees for patients stratified by age groups or by urine protein levels within the primary and secondary kidney disease cohorts.

In the primary kidney disease cohort, both age group and urine protein level were key factors in categories with particularly high costs. As shown in Figure 1, the oldest age group (14–18 years) incurred the highest costs, reaching 16,706 CNY (*F* = 4.80, *P* = 0.009). This amount was significantly greater than the costs for the groups aged 3–5 years (*P* = 0.040) and 6–13 years (*P* = 0.005) on *post-hoc* comparisons. Additionally, the 1 + urine protein level group had significantly higher medical costs, averaging 29,813 CNY (*F* = 2.62, *P* = 0.035), and in *post-hoc* comparisons, the costs for this group were significantly higher than those for the other four urine protein level groups (*P* = 0.003, 0.005, 0.004, and 0.011, respectively).

In the secondary kidney disease cohort, no significance differences in costs were observed among the age groups (*F* = 0.54, *P* = 0.586). According to the urine protein level though, the 3 + urine protein level group incurred significantly higher medical costs than the other groups, averaging 62,841 CNY (*F* = 7.19, *P* < 0.001), and on *post-hoc* comparisons, the costs for this group were significantly higher than the costs for each of the other four groups (all *P* < 0.001).

### 3.3 Factors affecting medical costs for CKD patients

Table 3 presents the results of the multiple linear regression analysis conducted within the primary kidney disease cohort. With adjustments for age, sex, urine protein level, and length of hospital stay, total medical costs (in-hospital total fees) did not differ significantly between the groups with and without infection (*P* = 0.250). However, the model still revealed significant associations between patient age, urine protein level, length of hospital stay, and total medical costs (all *P* < 0.05). Overall, for each additional year of age, the average medical costs increased by 771.40 CNY. Moreover, each additional day of hospitalization added 684.20 CNY to the total cost. Patients with the 1 + urine protein level in this cohort incurred an average additional total medical cost of 13,442.22 CNY compared with those with negative urine protein, while for patients with the 3 + urine protein level, the total medical cost was on average 5,753.90 CNY less than that for patients with negative urine protein.

**Table 3:** Multiple linear regression analysis of factors influencing in-hospital total fees within the primary kidney disease cohort.

Parameters	B		95% CI		*t*	*P*
	Estimate	S.E.	Lower	Upper		
Infection (overall)	2,471.90	2,144.31	−1,746.57	6,690.37	1.15	0.250
Age	771.40	189.67	398.26	1,144.54	4.07	<0.001
Sex						
Male	Reference	–	–	–	–	–
Female	−1,413.42	2,253.47	−5,846.65	3,019.80	−0.63	0.531
Urine protein level						
Negative	Reference	–	–	–	–	–
A small amount	−3,225.14	4,428.48	−11,937.25	5,486.97	−0.73	0.467
1+	13,442.22	5,694.63	2,239.24	24,645.20	2.36	0.019
2+	−4,517.77	2,793.10	−10,012.61	977.06	−1.62	0.107
3+	−5,753.90	2,833.37	−11,327.95	−179.85	−2.03	0.043
Hospital stay	684.20	100.25	486.99	881.42	6.83	<0.001

CI, confidence interval; S.E., standard error.

The multiple linear regression analysis results for the secondary kidney disease cohort are presented in Table 4. As indicated, patients in the infection group, on average, incurred an additional expenditure of 13,572.55 CNY compared with those in the non-infection group (*P* = 0.001). Of the covariates, each additional day of hospitalization resulted in an extra cost of 1953.23 CNY (*P* < 0.001). Patients with the 3 + urine protein level in this cohort incurred an average additional total medical cost of 20,360.05 CNY relative to patients with negative urine protein (*P* = 0.008).

**Table 4:** Multiple linear regression analysis of factors influencing in-hospital total fees within the secondary kidney disease cohort.

Parameters	B		95% CI		*t*	*P*
	Estimate	S.E.	Lower	Upper		
Infection (overall)	13,572.55	4,226.02	5,253.35	21,891.75	3.21	0.001
Age	1,060.27	573.17	−68.07	2,188.60	1.85	0.065
Sex						
Male	Reference	–	–	–	–	–
Female	3,449.46	4,954.93	−6,304.64	13,203.57	0.70	0.487
Urine protein level						
Negative	Reference	–	–	–	–	–
A small amount	−518.19	5,726.00	−11,790.20	10,753.81	−0.09	0.928
1+	−12,205.71	6,986.40	−25,958.90	1,547.47	−1.75	0.082
2+	−8,081.25	5,074.85	−18,071.43	1,908.92	−1.59	0.112
3+	20,360.05	7,662.30	5,276.31	35,443.80	2.66	0.008
Hospital stay	1,953.23	144.29	1,669.19	2,237.28	13.54	<0.001

## 4 Discussion

Research to date regarding the hospitalization costs and specific components for pediatric patients with CKD has been limited. Unlike adult patients, children with CKD rarely present with comorbidities such as hypertension, diabetes, or coronary heart disease; instead, infection has become a prevalent complication, particularly since the onset of the COVID-19 pandemic (9, 13). The present study included 721 pediatric CKD patients and revealed that hospitalization costs vary significantly based on infection status, age, and CKD classification (primary vs. secondary kidney disease). Patients with infections incurred substantially higher hospitalization costs than those without, primarily due to longer lengths of stay and increased medical service expenses. Additionally, age and urinary protein level emerged as critical determinants of hospitalization costs, with older patients and those with higher urinary protein levels facing greater financial burdens. These findings underscore the urgent need for effective infection management to alleviate economic pressures on families and the healthcare system, while also supporting targeted interventions based on identified risk factors.

The economic burden associated with infection in pediatric CKD patients is significant, and our findings align with the existing literature, which has consistently identified infection as a major complication in this population (14). Infections can worsen the progression of CKD, leading to further kidney damage and increasing the risk of severe complications such as sepsis. The associated decline in health often results in longer hospital stays, which directly increases hospitalization costs. Multiple studies (15, 16) have shown that the occurrence of infection substantially contributes to elevated medical expenses, placing additional financial strain on families and healthcare systems. Moreover, recurrent infections can negatively impact a child's overall health and development, resulting in missed educational opportunities and increased psychological stress. The results of our present study underscore the stark cost differences between cases with and without infection, emphasizing the urgent need for the development and implementation of effective infection prevention strategies. Targeted interventions aimed at reducing infection rates are crucial for alleviating these economic burdens and improving patient outcomes (17).

In the present study, age was identified as a critical factor influencing hospitalization costs for pediatric CKD. Age is known to significantly impact the progression of CKD due to physiological changes in kidney function and the accumulation of comorbidities over time. Younger patients may experience different disease manifestations and treatment responses than older patients, whose kidneys may already be compromised by age-related factors and other health issues. Our findings indicate that adolescents aged 14–18 years incur significantly higher expenses than younger children, likely due to the complexities of managing CKD in this age group, which often faces unique psychosocial challenges and issues related to treatment adherence (18, 19). Effective management of pediatric CKD must address these age-specific challenges through tailored strategies, such as counseling on treatment adherence and providing psychosocial support to enhance overall care.

The present study also identified urinary protein level as a significant influencing factor for hospitalization costs in pediatric CKD patients. Urinary protein is well established as a critical biomarker for assessing kidney damage and disease progression in CKD. Elevated proteinuria is associated with deteriorating kidney function and an increased risk of cardiovascular complications, negatively affecting overall prognosis (20–22). Effective monitoring and management of urinary protein can provide valuable insights into disease severity and inform treatment strategies. The identification of urinary protein level as a significant determinant of hospitalization costs aligns with the understanding that higher proteinuria often indicates more severe disease, leading to greater healthcare utilization. However, it is important to note that severe proteinuria may be caused by systemic infections, complicating the relationship between proteinuria and hospitalization costs. While controlling proteinuria is crucial for long-term disease outcomes, it is debatable whether it alone can significantly reduce hospitalization costs. Factors such as infection management and complications also play critical roles in influencing hospitalization expenses. Therefore, comprehensive strategies addressing multiple aspects of CKD management may be more effective for reducing costs (20).

The findings of the present study can significantly inform clinical practice for the management of pediatric CKD. A multidisciplinary approach including nephrologists, pediatricians, and infectious disease specialists is essential for enhancing patient outcomes while mitigating the economic burdens of treatment. This collaborative care model can address the multifaceted health needs of the children by focusing not only on disease management but also on psychosocial support. Consistently, previous studies have found that integrating different specialties improves treatment adherence and overall health outcomes in pediatric populations facing complex health challenges (3, 23). Additionally, the identification of high-risk groups, particularly infants who may transition from acute kidney injury to chronic disease, allows for timely intervention (24). Early identification through routine screening for urinary tract anomalies in newborns can significantly prevent CKD progression and reduce long-term healthcare costs (25). Furthermore, the implementation of family education programs that emphasize infection prevention and management can empower families to not only avoid some infections but also to recognize symptoms and seek timely medical attention when infections do occur, thereby reducing hospitalization risks and associated expenses. This proactive approach will not only enhance clinical outcomes but also foster a supportive environment for families navigating the complexities of pediatric CKD.

From a healthcare policy perspective, the findings of the present study underscore the necessity for targeted resource allocation to enhance infection prevention programs specifically for pediatric CKD patients. Policymakers should prioritize funding initiatives focused on vaccinations, early screening, and educational efforts for families and caregivers, as effective programs will raise awareness about infection prevention and potentially reduce infection rates and related hospitalization costs (26, 27). Additionally, the development of cost-effective care pathways that incorporate preventive measures would help to optimize healthcare expenditures while improving patient outcomes. In recent years, Diagnosis-Related Groups (DRGs) have emerged as a significant payment method in China's healthcare system, referring to a model in which providers are reimbursed based on specific procedures or diagnoses rather than the length of hospital stay (28, 29). A data-driven approach to refining DRG classifications, based on the cost structures identified in this study, can improve reimbursement models for healthcare providers. By pinpointing high-cost factors, healthcare systems can implement preventive strategies, enabling more accurate assessment of pediatric CKD hospitalization costs. This approach will reduce the financial strain on families while strengthening healthcare infrastructure, and ensuring better long-term care for pediatric CKD patients.

This study provides valuable insights but has several limitations. First, the retrospective design may introduce selection bias, as only hospitalized patients were included, potentially omitting cases managed successfully on an outpatient basis. Future studies should incorporate outpatient data for a more comprehensive analysis of the economic burden of pediatric CKD. Additionally, most CKD cases in the study cohort were stages 1–3, which is typical in pediatric populations, and this early-stage predominance may have skewed the economic burden analysis. The use of a single-center database also limits the generalizability of the findings, as regional differences in medical practices, resources, and patient populations could influence the results. Future research should include multi-center data and longitudinal studies to better understand the long-term economic impact and the effectiveness of intervention strategies in diverse healthcare settings.

## 5 Conclusion

In conclusion, the results of the present study underscore the significant economic burden of hospitalization among pediatric CKD patients, with infection, age, and urinary protein level serving as critical determinants of costs. Our findings indicate that patients who experience infection incur markedly greater hospitalization expenses, primarily due to longer stays and increased medical service fees. Our analysis revealed that among different age groups, adolescents face the highest costs, highlighting the need for age-specific management strategies that address unique challenges commonly faced by different age groups. Moreover, the association between a higher urinary protein level and increased healthcare utilization emphasizes the importance of effective interventions to manage this condition. A multidisciplinary approach involving nephrologists, pediatricians, and infectious disease specialists is essential for improving patient outcomes while reducing the financial strain on families. Policymakers should prioritize funding for infection prevention and educational initiatives for families to enhance care and reduce hospitalization rates. Overall, addressing these economic challenges is crucial for both supporting families and improving the quality of care for children with CKD. Future research should expand to include outpatient data to achieve a more comprehensive understanding of the economic impact of pediatric CKD and inform the development of effective intervention strategies.

## Data Availability

The original contributions presented in the study are included in the article/Supplementary Material, further inquiries can be directed to the corresponding authors.
